# Targeted Antibiotic Prophylaxis for Lung Transplantation in Cystic Fibrosis Patients Colonised with *Pseudomonas aeruginosa* Using Multiple Combination Bactericidal Testing

**DOI:** 10.1155/2012/135738

**Published:** 2012-07-16

**Authors:** Helmy Haja Mydin, Paul A. Corris, Audrey Nicholson, John D. Perry, Gerard Meachery, Emma C. L. Marrs, Steven Peart, Christine Fagan, James L. Lordan, Andrew J. Fisher, Frances K. Gould

**Affiliations:** ^1^Institute of Transplantation, The Freeman Hospital, Newcastle upon Tyne NHS Hospitals Trust, High Heaton, Newcastle upon Tyne NE7 7DN, UK; ^2^Institute of Cellular Medicine, Newcastle University, Newcastle upon Tyne NE2 4HH, UK; ^3^Department of Medical Microbiology, The Freeman Hospital, Newcastle upon Tyne NHS Hospitals Trust, High Heaton, Newcastle upon Tyne NE7 7DN, UK

## Abstract

Early infection is a recognised complication after lung transplantation in patients with cystic fibrosis (CF). Our centre uses multiple combination bactericidal testing (MCBT) when determining appropriate peritransplant prophylactic regimens. To evaluate our strategy, we compared the incidence of posttransplant infection in patients whose peritransplant antimicrobial regimens were determined using MCBT versus standard sensitivity testing. Patients with CF who were infected with *Pseudomonas aeruginosa* and underwent lung transplantations between 2000 and 2010 were included. Data was collected from clinical records and our microbiology database. Microorganisms cultured were mapped against antibiotic resistance, method of sensitivity testing, and antibiotics administered peritransplant. 129 patients were identified (mean age 28, male : female, 63 : 66). Fifty patients (38.8%) had antibiotics determined by MCBT. Two patients in the MCBT group developed septicaemia, 13 in the conventional group (*P* ≤ 0.05, 2-tailed Fisher's test). Sepsis was attributable to *P. aeruginosa* in one patient from the MCBT group and seven patients in the conventional group (*P* = 0.15). *P. aeruginosa* was recovered from the posttransplant pleural fluid of one patient who received MCBT-guided prophylaxis, six patients in the conventional group (*P* = 0.25). Patients given antibiotics based on MCBT had significantly lower rates of septicaemia and lower rates of empyema.

## 1. Introduction

Lung transplantation is an established therapeutic option for patients with advanced cystic fibrosis (CF) [1]. The success of the first transplant for a patient with CF in 1983 [2] spurred further refinement of the management and selection criteria of patients, leading to significant survival benefit [3]. However, patients with advanced CF present a unique microbiological challenge, with disease characterised by bronchiectasis, severe airflow obstruction, high bacterial loads, and recurrent lower respiratory tract infections [4–6]. Unsurprisingly, some centres turn down patients on the basis of colonisation with multiresistant bacteria. The most common respiratory pathogen that colonises patients with CF is *Pseudomonas aeruginosa*, with up to 80% of patients being culture-positive for this organism [7]. As the presence of this microorganism has negative prognostic implications [8], it is disturbing to note that levels of resistance to frontline antipseudomonal agents are very high [9].

The presence of multi- and pan-resistant *P. aeruginosa *renders methods of single-agent antibiotic susceptibility testing suboptimal [10]. The conventional manner of managing patients who are colonised with these microorganisms is to empirically treat them with combinations of antibiotics in the peritransplant period. Unfortunately, an empirical approach might lead to inadequate bactericidal levels and the prescription of antibiotics that antagonise each other [11]. This approach is undesirable, as patients with pan-resistant bacteria have shorter follow-up periods and decreased survival rates after transplant [12]. 

An alternative approach is to utilise Multiple Combination Bactericidal Testing (MCBT), a technique that had previously been used to systematically test bacterial isolates against multiple combinations of antibiotics to determine susceptibility patterns and identify optimal combinations for potential treatment [13]. Previous studies have demonstrated that using combinations of antimicrobials may generate higher levels of *in vitro *bactericidal activity against *P. aeruginosa *[13] and *Burkholderia cepacia *complex [14]. 

Our centre currently advocates the use of MCBT (with modified antimicrobial concentrations) to determine appropriate prophylactic regimens in patients about to undergo lung transplantation for CF and other lung pathologies that are colonised with antibiotic-resistant Gram-negative bacteria. To evaluate the effectiveness of our strategy, we undertook a retrospective analysis to compare the rates of posttransplant infection in patients whose peritransplant antimicrobial regimens were determined using the MCBT versus those who had their antibiotics chosen via conventional sensitivity testing. 

## 2. Methods

### 2.1. Case Review

We performed a retrospective analysis of all patients who underwent lung transplantation for CF between January 2000 and August 2010. Patients were included in the review if they were colonized pretransplant with *P. aeruginosa* (as demonstrated by sequential sputum cultures) and were excluded if they were colonized with *B. cepacia* complex. Data was collected from patients' case notes and clinical charts; looking specifically at incidences of septicaemia at 30 days, posttransplant wound infection at 30 days, empyema at 30 days, all-cause mortality at 30 days, and all-cause mortality at one year. These were mapped against antibiotic resistance, method of sensitivity testing, and choice of antibiotics that were administered. Statistical differences were calculated using two-tailed Fisher's test. 

### 2.2. Infection Definitions

Our microbiology database was interrogated to identify microorganisms cultured from these patients. Infections were defined by combining positive laboratory culture from tissue source (blood, surgical wound, and pleural fluid) with at least two of the following four parameters:

- tachycardia (heart rate >90 beats per minute),
- hypotension (systolic blood pressure <90 mmHg),
- body temperature <36°C or >38°C,
- abnormal inflammatory markers (white cell count <4 × 10^9^ cells/L or >12 × 10^9^ cells/L, C-reactive protein >10 mg/L).

### 2.3. Multiple-Combination Bactericidal Testing

Prior to 2001, prophylactic antimicrobials were chosen based on disc susceptibility testing using British Society for Antimicrobial Chemotherapy (BSAC) breakpoints [15]. If sputum was culture positive for pan-resistant *P. aeruginosa* or recent culture results were unavailable, patients were treated empirically with aztreonam, an antistaphylococcal agent (either flucloxacillin or clindamycin), and metronidazole. Patients who did not receive peritransplant antibiotics chosen via the MCBT method were deemed to have received antibiotics using “conventional” means. Patients in both MCBT and conventional groups had antibiotics commenced at induction. Perioperative antibiotics were continued until the patient was extubated and could demonstrate a good cough, (two-three days) to a maximum of seven days.

We introduced the MCBT at our centre in 2001. This was used in conjunction with conventional means of choosing peritransplant antibiotics, but, since 2008, MCBT became the default method for the determination of bactericidal agents for all patients colonised with antibiotic-resistant Gram-negative nonfermenters. Bactericidal activity was determined by testing at least 12 antimicrobials individually and in combination with each other, leading to 66 different combinations. Several morphotypes of *P. aeruginosa* from at least two pretransplant sputa were selected for testing. 

Each antimicrobial or combination of antimicrobials was tested in IsoSensitest broth using systemic breakpoint concentrations as specified by the BSAC. After 48 hours incubation at 37°C, the turbidity of each broth was measured at 620 nm. Broths without detectable bacterial growth were subcultured onto blood agar to calculate 99.9% bacterial kill [13]. Peritransplant antibiotic regimens were then chosen based on MCBT results and patients' allergy history. Any other Gram-negative species (including nonfermenters and/or Enterobacteriaceae) that may have isolated alongside *P. aeruginosa* were also tested using the MCBT, and antibiotic cocktails were chosen that showed bactericidal activity against such mixtures of species. 

### 2.4. Peritransplant Immunosuppression Therapy

Our centre used a three-day induction protocol with antithymocyte globulin (titrated by flow cytometric analysis of peripheral blood T lymphocytes) and intravenous methylprednisolone at a dose of 2 mg/kg. Patients were given triple immunosuppression posttransplant (azathioprine, ciclosporin and prednisolone). Ciclosporin was commenced after transplant as soon as renal function was deemed satisfactory. Alternatives were used in the context of an international clinical trial (mycophenolate) or in cases of ciclosporin intolerance (tacrolimus was used). Up to five days of intravenous ciclosporin was given in the context of poor ciclosporin absorption in patients with CF. 

## 3. Results 

Between January 27, 2000 and August 23, 2010, 163 lung transplants were performed on patients with CF. This number included patients who were previously turned down by other centres on the basis of their microbiology. A total of 129 patients were colonized with *P. aeruginosa *and not colonized with *B. cepacia *complex. Mean age was 28 years old. There were 63 male patients and 66 female patients. Fifty patients were given antibiotics that were chosen based on the MCBT, and there were 79 patients in the conventional group. Patient characteristics are listed in Table 1.

Our patients were colonized with strains of *P. aeruginosa* with varying degrees of antibiotic resistance. We defined pan-resistance as resistance to antipseudomonal quinolones, *β*-lactams, aminoglycosides, and colomycin. In this cohort, two patients were colonised with pan-resistant *P. aeruginosa. *These organisms were resistant to all single agents tested but bactericidal combinations with colomycin and another agent were identified. Multiresistant organisms were defined as resistant to three out of the four antimicrobial groups. Seventy-one patients were colonised with multiresistant *P. aeruginosa,* and nine patients were colonised with fully susceptible strains. Forty-seven patients were colonised with organisms that were resistant to one or two groups of antimicrobials. 

**Figure 1:**
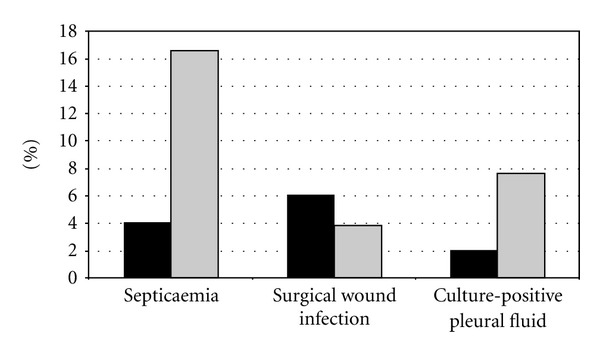
Proportion (%) of patients with infectious complications (at 30 days) after lung transplantation. Black bars: MCBT group. Grey bars: conventional therapy group.

Figure 1 shows the relative rates of infectious complications after lung transplantation in both groups. Two patients (4%) who were given antibiotics based on MCBT developed septicaemia compared to 13 (16.5%) in the conventional group (*P* ≤ 0.05). The occurrence of Gram-negative sepsis was statistically lower in the MCBT group, and *P. aeruginosa* was responsible for only one case of septicaemia compared with seven in the conventional therapy group (see Table 2). *P. aeruginosa* was recovered from the posttransplant pleural fluid of one patient (2%) in the MCBT group, as opposed to six (7.6%) in the conventional group (*P* = 0.25). There were no statistically significant differences in the rates of surgical wound infection (6% in the MCBT group, 3.8% in the conventional group). There were no statistically significant differences in all-cause mortality rate at 30 days, with a 10% mortality rate in the MCBT cohort and 6.33% in the conventional group. This lack of statistical significance was replicated in all-cause mortality rate at one year (22% in the MCBT group, 19% in the conventional group). 

## 4. Discussion

Lung transplantation for CF accounts for approximately one-third of all single sequential lung transplants performed at our centre [16]. The complex microbiology involved in this cohort of patients may lead to anxiety when listing patients with multi- and pan-resistant *P. aeruginosa*, but this paper demonstrates for the first time that the MCBT may have a significant role in altering posttransplant infective complications for patients with CF. The data indicates that whilst there may not be evidence for an effect on all-cause mortality, patients who had antibiotics chosen using MCBT had lower rates of morbidity. The presence of pleural infection and septicaemia not only negatively impacts the patients' transplant journey but prolongs the hospital length-of-stay and adds substantial economic burden [17]. 

We had excluded patients who were colonised with *B. cepacia* complex for the purposes of this retrospective analysis. This is due to the tendency of these patients to succumb to overwhelming sepsis with one-year mortality rates of between 50% and 100% [18, 19]. Our group had also previously noted that infection with *Burkholderia cenocepacia* in particular led to even poorer mortality outcomes [20]. 

Unfortunately, we were unable to look at exact causes of death (septicaemia in particular) in our patient cohort as causes of death were not readily identifiable in all cases. This is a reflection of the wide geographical referral area that is covered by our centre as well as the degree of shared care with referring centres (covering the north of England, Scotland, Northern Ireland, and the Republic of Ireland). A previous analysis of all patients who underwent lung transplantation for CF at our centre [16] had identified sepsis as the cause of death in 18 cases (26% of all recorded CF transplantation recipient deaths). *B. cepacia* complex was implicated in seven of these cases. In an additional three cases, clinical sepsis was recognised without identification of an underlying pathogen. Prospective studies would play an important role in determining if the use of MCBT can significantly decrease the rate of early mortality due to septicaemia. 

Aaron et al. had compared the efficacy of using antimicrobial combinations derived from MCBT with those derived from conventional susceptibility testing for treatment of acute pulmonary exacerbations of CF. They concluded that regimens based on MCBT results did not result in a better clinical or bacteriological outcome [21]. It is difficult to make comparisons between our findings and theirs for both clinical and technical reasons. On the clinical side, the patient populations (post-lung-transplant CF versus non-lung-transplant CF) and outcome measures were different. There are also major differences in the antimicrobial concentrations used in our modified MCBT test and the test as originally described [13, 14]. 

In our study, systemic breakpoint concentrations specified by the BSAC to define susceptibility were used in a modified MCBT whereas antimicrobial concentrations in the original MCBT “were chosen on the basis of published estimates of the average peak levels seen in serum after standard single-dose intravenous administration” [13]. As a result, the antimicrobial concentrations used in our modified MCBT were typically two- to eightfold lower than those previously described [14]. As a consequence, fewer isolates would be likely to be classified as susceptible in our study.  Table 3 shows the antimicrobials used in this study with their corresponding concentrations. It also compares these with those chosen by Aaron et al. [14]. It is impossible to conclude whether the use of lower breakpoint concentrations of antimicrobials would have led to more favourable outcomes in the study of Aaron et al. [21]. 

As this is a retrospective study, we readily acknowledge that there are inherent limitations to our findings. The ten-year period covered by the study has seen numerous changes with regards to developments in lung transplantation. These include changes in immunosuppression regimen and the natural progression with regards to peritransplant management as the members of our centre gain more experience. Given the nonrandomized nature of this study and temporal confounding factors, we cannot definitely conclude that MCBT should be given at induction to all patients colonized with *P. aeruginosa*. However, the fact that we are dealing with a unique cohort of patients with significant microbiological challenges might render it unethical to conduct a full randomized control trial. Furthermore, it is unlikely that sufficient numbers are recruited to adequately power such a study.

The data presented is the first indication that patients given antibiotics based on MCBT results had significantly lower rates of septicaemia and lower rates of positive microbiological cultures in their pleural effusions. This is an encouraging finding, lending credence to the need for multi-centre prospective studies to be performed that will ideally lead to no patients being turned down for a lung transplant on the basis of colonisation with resistant microorganisms.

## Figures and Tables

**Table 1:** Patient characteristics.

Characteristic	MCBT	Conventional	Overall
Total number of transplants on patients with cystic fibrosis	65	98	163
Patients included in the study	50	79	129
Male : female	23 : 27	40 : 39	63 : 66
Mean age (range)	28.7 (16–53)	27.3 (15–54)	28 (15–54)
Single sequential lung transplant	49	78	127
Liver-lung transplant	1	0	1
Heart-lung transplant	0	1	1

**Table 2:** Causes of post-lung-transplant sepsis.

	Conventional group (*n* = 79)	MCBT group (*n* = 50)	*P*
Total cases of sepsis	13^a^	2^b^	0.046
Gram-negative sepsis	10	1	0.049
*P. aeruginosa *sepsis	7	1	0.15
Gram-positive sepsis	3	1	1.0

^a^ *P. aeruginosa* (*n* = 7), *Serratia marcescens* (*n* = 2), *Achromobacter xylosoxidans* (*n* = 1), *Enterococcus *spp. (*n* = 2), *Candida glabrata* (*n* = 1).

^b^ *P. aeruginosa* (*n* = 1), *Staphylococcus aureus* (*n* = 1).

**Table 3:** Antimicrobial agents and breakpoint concentrations (mg/L) used in the Multiple Combination Bactericidal Test.

Antibiotic	Modified MCBT using BSAC breakpoints	Original MCBT study Aaron et al. [14]
Amikacin	NT	32
Aztreonam	8	32
Colomycin	4	NT
Ceftazidime	8	32
Chloramphenicol	8	20
Trimethoprim-sulfamethoxazole	10/2	10/2
Ciprofloxacin	1	2
Doripenem	8	NT
Fosfomycin	64	NT
Imipenem	NT	10
Meropenem	4	32
Minocycline	NT	2
Piperacillin-tazobactam	16/2	32/4
Ticarcillin-clavulanate	16/1	32/10
Temocillin	8	NT
